# Evaluation of Students' Proficiency in Removing Gutta-Percha Prior to Fiber Post Placement: A Retrospective Study

**DOI:** 10.7759/cureus.57203

**Published:** 2024-03-29

**Authors:** Arwa Bafail, Amnah A Algarni, Jameel A Abuljadayel, Rayan A Hawsawi, Mahmoud Alsulaimani, Shadan Sharbib, Abdulmajeed Baik, Hatem H Hamadallah, Mahir A Mirah

**Affiliations:** 1 Restorative Dental Sciences Department, College of Dentistry, Taibah University, Madinah, SAU; 2 Preventive Dentistry Department, College of Dental Medicine, Umm Al-Qura University, Makkah, SAU; 3 Restorative Department, College of Dental Medicine, Umm Al-Qura University, Makkah, SAU; 4 Department of Preventive Dental Sciences, College of Dentistry, Taibah University, Madinah, SAU; 5 Dental School, College of Dentistry, Taibah University, Madinah, SAU

**Keywords:** radiograph, students proficiencies, post and core, fiber post, gutta-percha

## Abstract

Introduction: This retrospective study focuses on dental students’ proficiency in removing gutta-percha during fiber post space preparation, which is important for maintaining apical seal integrity in endodontically treated teeth. Emphasizing the significance of preventing further reinfection. The primary objective of this study was to assess the proficiency of undergraduate dental students in the manipulation of gutta-percha during fiber post preparation, specifically focusing on the psychomotor skills involved. In addition, the study aims to determine the predictive value of these skills on the ultimate clinical outcomes of the procedure, with particular emphasis on variations attributable to tooth type.

Materials and methods: The analysis encompassed 290 periapical radiographs obtained from endodontically treated teeth, all of which had undergone fiber post placement by undergraduate students at the College of Dentistry, Taibah University, Saudi Arabia. Postoperatively, the length of the remaining gutta-percha was measured by two experts in the field, and subsequent outcomes were classified into three categories: optimal, suboptimal, and inadequate, based on the extent of gutta-percha remaining.

Results: Students showed optimal removal rates ranging from 33.3% to 73.1%. Among the studied sample, upper anterior teeth were the highest included teeth (n=117, 40%). Remarkably, lower anterior teeth were more prone to suboptimal and inadequate gutta-percha remaining lengths (n=24, 33%). However, the chi-square test revealed no statistically significant difference in students’ psychomotor skills while removing the gutta-percha and preparing the teeth to receive fiber posts across tooth types (p-value > 0.05).

Conclusion: With the limitations of this study, more than half of the included cases show that undergraduate students of the College of Dentistry at Taibah University, Saudi Arabia, possess an optimum level of psychomotor skills in removing gutta-percha while preparing the teeth for receiving fiber posts. This study suggests enhancing the preclinical training of students by considering more training on different tooth types in relation to dental arches.

## Introduction

Root canal treatment is one of the main aspects of dentistry that preserves teeth that suffer from pain or infection in the root canals. This treatment involves the use of gutta-percha (GP) to fill the root canal space. Endodontically treated teeth are often significantly damaged, compromising their structural integrity. Insufficient support for a permanent restoration can indicate intracanal post placement.

Preparing the post space involves the removal of the GP from the coronal and middle parts of the root canal. An effective apical seal prevents root canal reinfection by penetrating the bacteria and infectious material into the root canal space from the periapical space [1-4]. Therefore, it is necessary to be careful while preparing the root canal space to avoid compromising the apical seal. The length of the remaining GP to withstand bacterial penetration and maintain the apical seal should be between 3 and 5 mm at the apex of the root [5,6]. A residual apical GP length of 3 mm is associated with a higher incidence of periapical radiolucency than roots with a longer residual root canal filling [7].

Beyond critical thinking, mastering this procedure can ensure excellence in education standards and patient care quality [8]. Therefore, it is recommended that the practical component of the curriculum and preclinical laboratory should prepare the student with the best clinical skills. Therefore, the curriculum’s efficiency should be evaluated to ensure that clinical outcomes align with educational outcomes [9].

A few studies have assessed the length of the remaining GP after post placement [3-5,10]. However, no studies have evaluated student performance in this procedure using maxillary and mandibular teeth across different types of teeth. This study assessed the radiographic length of the remaining GP following post space preparation. This study focuses on students’ proficiency in identifying common mistakes and proposes targeted educational strategies to enhance the quality of endodontic education and treatment outcomes.

## Materials and methods

The study included periapical radiographs of endodonticly treated teeth with GP and fiber post. Inclusion criteria were teeth with fiber posts performed by undergraduate students and interns with clear periapical postoperative radiographs with no procedural errors such as perforation. Exclusion criteria were teeth with fiber post performed by postgraduate students or dentists, teeth with procedural errors, and unclear periapical postoperative radiograph.

Between January and March 2024, 290 periapical radiographs were collected from the R4 system at the College of Dentistry. Two consultants independently measured the remaining GP using the postoperative periapical radiograph. When discrepancies emerged between them, a third consultant was invited to reach a consensus. The distance from the coronal end to the apical end of the GP was measured in millimeters and transferred to an Excel sheet.

The results were categorized into three groups: optimal (3-5 mm of remaining GP), suboptimal (>5 mm remaining), and inadequate (<3 mm remaining).

Data were analyzed using SPSS, version 13.0 (IBM Corp., Armonk, NY, USA), and descriptive statistics of the frequency of proficiency of students were tabulated. The proficiency levels among tooth types were compared using chi-square tests. A p-value of 0.05 was considered statistically significant.

The research protocol received ethical approval from the ethics committee of the College of Dentistry, Taibah University (TUCDREC/030324/MAMirah).

## Results

The proficiency of dental students in the removal of GP was measured in different types of teeth. The remaining GPs were categorized into three levels: inadequate, optimal, and suboptimal, as clearly indicated in Table 1.

**Table 1:** Proficiency of students in removing gutta-percha (GP) by tooth type Optimal (3-5 mm of remaining GP), suboptimal (>5 mm remaining), and inadequate (<3 mm remaining). The P-value represents the statistical significance differences between the remaining GP across different types of teeth, as determined by the chi-square test. A P-value 0.05 is considered statistically significant.

Teeth Type	Inadequate	Optimal	Suboptimal	Total	P-value
Upper anterior	7 (6%)	68 (58.1%)	42 (35.9%)	117	0.396
Upper premolar	6 (12.2%)	27 (55.1%)	16 (32.7%)	49	
Upper molar	1 (7.1%)	8 (57.1%)	5 (35.7%)	14	
Lower anterior	7 (19.4%)	12 (33.3%)	17 (47.2%)	36	
Lower premolar	3 (6.3%)	30 (62.5%)	15 (31.3%)	48	
Lower molar	1 (3.8%)	19 (73.1%)	6 (23.1%)	26	
Total	25 (8.6%)	164 (56.6%)	101 (34.8%)	290	

The remaining GP of the upper anterior teeth was measured in 117 cases. It was revealed that after investigating the recordings, approximately 6% (n=7) were deemed inadequate, 58.1% (n=68) were optimal, and 35.9% (n=42) were suboptimal. In total, 12.2% (n=6) of the premolars were categorized as inadequate, 55.1% (n=27) were categorized as optimal, and 32.7% (n=16) were categorized as sub-optimal. In terms of the upper molars (n=14), 7.1% (n=1) performed inadequately, 57% (n=8) were determined to be at optimal levels, and 35.7% (n=5) were inadequate.

Among the lower anteriors, 19.4% (n=7) of the 36 cases were inadequate, 33.3% (n=12) was optimal, and 47.2% (n=17) were suboptimal. Lower premolar, demonstrated in 48 cases, 6.3% (n=3) inadequate, 62.5% (n=30) optimal, and 31.3% (n=15) suboptimal. Optimal GP length of lower molars was found in 3.8% (n=1) of patients, 73.1% (n=19) had optimal proficiency and 23.1% (n=6) had suboptimal removal skill.

In general, there was an obvious difference between the varieties of effective removal grade of the students on different teeth, which ranged from 33.3% to 73.1%. The total observation constituted 290 individuals with 8.6% (n=25) labeled as inadequate, 56.6% (n=164) as optimal, and the rest from the same cohort (n=101) indicated as suboptimal.

The outcomes of the chi-square test did not exhibit any significant difference in the levels of proficiency among the teeth type (P=0.396).

## Discussion

One of the most common clinical procedures that general dentists can perform daily is post-preparation [3]. An effective apical seal is important to obtain a successful endodontic treatment, and it is crucial to remain intact during post space preparation. Therefore, the ideal remaining GP in the apical part of the canal should be between 3 and 5 mm to minimize the possibility of reinfection [5,6]. Previous studies have proven that an apical seal's minimum acceptable clinical length, even for short roots, should not exceed 3 mm [4,11]. A study performed by Meshni et al. [12] showed similar results, and the remaining GP was 3-5 mm in 55% and >5 mm in 29% of the treated cases. Other studies reported more than two-thirds of the cases achieved suboptimum (>5 mm), while around one-third showed optimal measurments (3-5 mm) of remaining apical GP [2,3,13]. These differences could be related to sample heterogenecity between the studies, including type of teeth included and students' academic levels. The dominant outcome among these studies, however, was that the length of the apical seal was more than 3 mm for all different types of teeth. This indicates that most students have the required skills and knowledge to perform such a complicated procedure, even though this occurs under supervision [14].

Maxillary incisors were the most common type of teeth included in this study, followed by premolars. This observation is consistent with some of the previous studies [2,13]. Different order was observed in other studies where premolars were the most restored teeth followed by anteriors [3,5,15]. Few cases in this study showed insufficient apical seal (<3 mm), and they were mainly found in the anterior and premolars. This could be because students usually start their first case in clinical training with single or bi-rooted teeth with wider and less curved canals. In addition, a pulp chamber in molar teeth can be used for retention, so it can be relatively short when a post is needed. while anterior and premolars and core retention mainly rely on post dimensions, optimum length (i.e., equal to clinical crown) is necessary to prevent tooth fracture [16]. Restoring endodontically treated teeth with short roots, such as the mandibular anterior, might be challenging. In such a case, the minimum apical seal could be compromised to increase the post length and enhance retention and resistance [17]. However, the apical seal must be considered even in these cases to avoid microleakage and failure of the endodontic treatment [18]. 

Moreover, the complexity of the root anatomy of posterior teeth could affect placement of posts due to tapering of root canals and severe curvatures, as reported in several studies [5,7,19]. Therefore, dentists tend to cut the GP before reaching the curvature to reduce the risk of perforations and stripping [3]. This explains the findings of this study, which showed that remaining GP less than 3 mm was observed in only two molars compared with other types of teeth in this study. This finding agrees with other studies at Jazan University by Meshni et al. [12] and at Qassim University by Mathar and Almutairi [15].

Upper and lower molars were less than 15% of the treated cases. This could be due to the larger pulp chamber of the molars that could be used for core retention rather than using post. Another reason why molars are more difficult to treat is the more complex anatomy of a multi-rooted tooth, so undergraduate students tend to treat single or double roots [20,21]. Furthermore, the biomechanical behaviors of anterior teeth are different from those of premolars and molars [22]. Maxillary anterior teeth are more subjected to horizontal and oblique forces, making them more susceptible to fracture than posterior teeth. The latter are more subjected to vertical forces, which could indicate less susceptibility [23]. This might necessitate more mechanical considerations when restoring incisors with minimal remaining tooth structure [24].

An in vitro study revealed that a higher rate of microleakage was observed when the apical GP seal was 3 mm compared with 4 and 5 mm. However, no statistical significance was found between 3 and 4 mm, supporting the clinical recommendation that 3 mm is the least clinically acceptable apical seal during the post placement procedure [7]. The increased percentage of microleakage at the 3 mm level could be attributed to the lower penetration of sealer material due to the lower diameter and density of dentinal tubules at the apical third of the canal [25].

Results from this study encourage faculty members and clinical supervisors to enhance students' training to be more conservative during post space preparation to maintain optimum pical seal. In addition to apical microleakage prevention, the quality of fiber post adhesion to apical radicular dentin decreases toward the apical third of the canal [26]. The histological nature of dentinal tibules in the apical third of the canal might impact the resin infiltration and quality of hybrid layer formation. Besides, the apical third is less accessible so there is less control over humidity and smear layer effect in this area of the root, which might increase risk of voids formation. This could negatively impact the bond strength between fiber-post and radicular dentin [27].

Restoring mutilated teeth, starting with endodontic treatment followed by post placement, is a complex procedure requiring extensive training within each field. Although the current study showed that many cases obtained an optimum apical seal, a considerable percentage still displayed inadequate measures. Students’ level of clinical skills could reflect the quality of preclinical training, as it is an essential part of the undergraduate dental curriculum [28]. Attempts have been made to improve the level of preclinical training of dental undergraduates before starting clinical sessions [29]. It has been recommended to start with artificial teeth during preclinical training for post and core techniques to establish students’ skills until they grasp the basic clinical principles of post space preparation and placement. Afterward, preclinical training proceeds with extracted natural teeth for more realistic simulation before progressing into clinical situations. Moreover, the development of 3D-printed teeth simulating the natural anatomy of the tooth displayed promising results as a feasible preclinical teaching method [30]. This method has been explored and proved to be effective for several dental procedures, including endodontic training and post space preparation. Regarding clinical practice, an increasing number of treated cases and practical experience have been reported to be the main factors in achieving dental undergraduates’ perceived competence in prosthodontics [31]. Therefore, improving preclinical training methods and increasing the number of cases during clinical sessions are recommended to enhance students’ clinical outcomes. 

The limitation of the study was that the academic level and gender of the students were not considered in the analyses. Typically, such a procedure has to be clinically performed by the students in the final two years of the curriculum. The level of education may impact the proficiency of the procedure, and it is recommended to be considered in further investigations. Furthermore, the assessment of the apical seal and quality of the post and core should be further expanded to incorporate other dental colleges in Saudi Arabia to compare and identify areas for improvement in the current dental education practice.

## Conclusions

With the limitations of this study, almost 57% of the included radiographs show that undergraduate students of the College of Dentistry at Taibah University, Saudi Arabia, possess an optimum level of psychomotor skills in removing gutta-percha while preparing the teeth for receiving fiber posts. The average of both inadequate and suboptimal performance was around 43%. Although there were no statistically significant differences among the included teeth types. The descriptive analysis suggests enhancing the preclinical training of undergraduate students by considering more training on different tooth types in relation to dental arches, which in turn will improve the quality of the final treatment outcomes of both the clinical root canal and restorative part. 
